# Identification of Variants of Uncertain Significance in the Genes Associated with Thoracic Aortic Disease in Russian Patients with Nonsyndromic Sporadic Subtypes of the Disorder

**DOI:** 10.3390/ijms25158315

**Published:** 2024-07-30

**Authors:** Irina A. Goncharova, Sofia A. Shipulina, Aleksei A. Sleptcov, Aleksei A. Zarubin, Nail R. Valiakhmetov, Dmitry S. Panfilov, Evgeniya V. Lelik, Viktor V. Saushkin, Boris N. Kozlov, Ludmila P. Nazarenko, Maria S. Nazarenko

**Affiliations:** 1 Research Institute of Medical Genetics, Tomsk National Research Medical Center, Russian Academy of Sciences, 10 Ushaika Str., Tomsk 634050, Russia; irina.goncharova@medgenetics.ru (I.A.G.); sofia.beliaeva@medgenetics.ru (S.A.S.); alexei.sleptcov@medgenetics.ru (A.A.S.); aleksei.zarubin@medgenetics.ru (A.A.Z.); nail.valiakhmetov@medgenetics.ru (N.R.V.); ludmila.nazarenko@medgenetics.ru (L.P.N.); 2 Cardiology Research Institute, Tomsk National Research Medical Center, Russian Academy of Sciences, 111a Kievskaya Str., Tomsk 634012, Russia; pand2006@yandex.ru (D.S.P.); eva00@list.ru (E.V.L.); saushkin.vv@ya.ru (V.V.S.); bnkozlov@yandex.ru (B.N.K.)

**Keywords:** sporadic thoracic aortic aneurysm, thoracic aortic disease genes, variants of uncertain significance

## Abstract

Nonsyndromic sporadic thoracic aortic aneurysm (nssTAA) is characterized by diverse genetic variants that may vary in different populations. Our aim was to identify clinically relevant variants in genes implicated in hereditary aneurysms in Russian patients with nssTAA. Forty-one patients with nssTAA without dissection were analyzed. Using massive parallel sequencing, we searched for variants in exons of 53 known disease-causing genes. Patients were found to have no (likely) pathogenic variants in the genes of hereditary TAA. Six variants of uncertain significance (VUSs) were identified in four (9.8%) patients. Three VUSs [*FBN1* c.7841C>T (p.Ala2614Val), *COL3A1* c.2498A>T (p.Lys833Ile), and *MYH11* c.4993C>T (p.Arg1665Cys)] are located in genes with “definitive” disease association (ClinGen). The remaining variants are in “potentially diagnostic” genes or genes with experimental evidence of disease association [*NOTCH1* c.964G>A (p.Val322Met), *COL4A5* c.953C>G (p.Pro318Arg), and *PLOD3* c.833G>A (p.Gly278Asp)]. Russian patients with nssTAA without dissection examined in this study have ≥1 VUSs in six known genes of hereditary TAA (*FBN1*, *COL3A1*, *MYH11*, *NOTCH1*, *COL4A5*, or *PLOD3*). Experimental studies expanded genetic testing, and clinical examination of patients and first/second-degree relatives may shift VUSs to the pathogenic (benign) category or to a new class of rare “predisposing” low-penetrance variants causing the pathology if combined with other risk factors.

## 1. Introduction

It is known that thoracic aortic aneurysm/dissection (TAAD) has a significant genetic component, with 20% of patients having a genetic cause of the disease [1]. The remaining 80% of cases have neither a family history of aortic disease nor causal pathogenic variants in the genes implicated in hereditary aneurysms and are considered sporadic subtypes of the disorder. The sporadic subtype is frequently defined as a multifactorial pathology because, along with genetic causes, it is characterized by the presence of a wide range of risk factors [2]. This subtype is characterized by high heterogeneity involving diverse genetic variants: from very rare with high penetrance to variants common in the population that were identified in genome-wide association studies (GWASs) and posing a lower risk of the disease [1,2,3,4].

Studies on nonsyndromic sporadic TAAD (nssTAAD) have shown the presence of rare pathogenic variants and common single-nucleotide polymorphisms (SNPs) in genes associated with Marfan syndrome (*FBN1*) and Loeys–Dietz syndrome (*TGFBR1* or *TGFBR2*) as well as rare copy number variants (CNVs) affecting *MYH11*, *ELN*, or *TGFB2*, whose mutations correlate with familial types of TAAD [1,5,6,7,8]. In patients with sporadic thoracic aortic dissection, rare pathogenic variants and CNVs have been identified in hereditary-aneurysm genes, including *FBN1*, *MYH11*, *EFEMP2*, *TGFBR2*, *FBN2*, *COL3A1*, and *MYLK*, and 28% of the patients have >1 variant of unknown significance (VUS) in the genes of hereditary aneurysms; this occurrence is significantly higher than in a control group [9,10,11].

A substantial number of molecular genetic studies combine TAAD together into one group because dissection is considered a natural consequence of an aneurysm [2,3,12,13,14,15,16]. Nevertheless, it has been reported that 60% of patients with type A dissection (according to the Stanford classification) have a diameter of <5.5 cm, and type B dissections occur without significant dilatation of the aorta and are thus suggestive of a possibly different molecular pathogenesis of aneurysm and dissection of the thoracic aorta [2,4]. Studies specifically examining ascending aortic aneurysm without dissection are rare [17].

There are several guidelines for the management of patients with syndromic or familial forms of TAAD, including genetic testing for mutations in certain genes [18,19]. At the same time, patients with nssTAAD have also clinically relevant variants in the genes of hereditary aneurysms, and some relatives of patients with aortic aneurysm have pathological changes in thoracic aorta images that can be discovered in a long-term (more than 10 years) follow-up. For example, in “sporadic” patients, this sign may indicate a de novo mutation, which subsequently may be classified as the etiology. Hence, guidelines for the management of patients with nssTAAD are being developed actively. These guidelines include recommendations for thoracic aorta visualization and genetic testing not only for patients but also for first- and second-degree relatives [2,20].

Currently, massive parallel sequencing is the most common technology for molecular genetic testing of patients with TAAD. Targeted panels for TAAD diagnostics include different numbers of genes for hereditary aneurysms in different ethnic groups [20,21,22,23]. In the Russian Federation, very few genetic studies, such as an analysis of specific exons of genes *ACTA2*, *NOTCH1*, and *FBN1* in patients with Marfan syndrome, have been performed on patients with thoracic aortic aneurysm [24,25,26].

There are also few articles that describe molecular genetic testing of patients with TAAD by exome/clinical exome sequencing [27,28,29]. The results indicate that exome sequencing is an effective approach to the identification of a genetic cause of TAAD and can be used in genetic counseling of patients and their families. Moreover, 10% of patients with syndromic and nonsyndromic thoracic aortic aneurysms share some mutations [20].

Genetic studies often combine familial and sporadic cases as well as aneurysms and aortic dissections; however, it is possible that each category has a unique spectrum of clinically significant variants. Besides, it is probable that there is an ethnic specificity for mutations in the genes of hereditary aneurysms [30].

Thus, the purpose of this study was to identify by massively parallel sequencing clinically relevant genetic variants in the genes of hereditary aneurysms in Russian patients with nssTAA without dissection.

## 2. Results

The study involved 41 Russian patients with sporadic ascending aortic nondissecting aneurysm. None of the patients were diagnosed with syndromic subtypes or had a family history of aortic dilatation or congenital connective tissue dysplasia. The majority of patients were males (65.8%) (Table 1).

The average age of the study group was 54 (48; 64) years and did not differ significantly between male and female participants (*p* = 0.559). The majority of patients (61.0%) had a bicuspid aortic valve. Arterial hypertension (64.3%), obesity (42.9%), hyperlipidemia (23.8%) and type 2 diabetes mellitus (T2DM) (7.1%) were also found in the study group. The prevalence of these risk factors did not differ between the male and female patients (Table 1). Coronary artery disease (CAD), angina pectoris, or myocardial infarction were found in 30.9% of patients and were more prevalent among males (*p* = 0.039). Atherosclerosis of coronary, carotid, renal or femoral arteries was detected in 50% of the patients and was represented mostly by coronary atherosclerosis (more often observed in males) (Table 1).

By computed tomography, we assessed the degree of aortic wall and coronary artery (CA) calcification in 26 patients with subsequent calculation of the Agatston index [31]. The average Ca-score in the group was as follows: CA, 71.2; aorta, 107. The Ca-score was higher in males than in females, although the difference was not statistically significant (Table 1).

As a result of clinical exome sequencing and identification of rare genetic variants in 53 genes of syndromic and hereditary TAA subtypes in Russian patients with nssTAA without dissection, no pathogenic or likely pathogenic variants were identified according to the American College of Medical Genetics (ACMG) classification. The 45 identified variants were classified as follows: 34 benign, 5 likely benign, and 6 VUSs (Supplementary Table S1).

Because no pathogenic or likely pathogenic variants were found in the study population, we provide a more detailed description of these VUSs (and their carriers) (Table 2). These variants may be associated with the pathology in question, but at present, this cause–effect relation is not proven. A detailed description of patients who are not VUS carriers is presented in Supplementary Table S3.

All identified VUSs are missense variants and were detected in genes of different categories according to the classification based on the strength of association with hereditary thoracic aortic aneurysms (HTAA) [13]: genes *FBN1*, *COL3A1*, and *MYH11* are in the first category (A) with a “definitive” effect of pathogenic variant on the pathology; *NOTCH1* belongs to the second category (B): “potentially diagnostic” genes that may be the cause of thoracic aortic dilatation but mostly correlate with other clinical signs; *COL4A5* is affiliated with the third category (C): genes with limited evidence of an effect on HTAA; and *PLOD3* is in the fourth category (D): genes associated with diseases of the thoracic aorta according to experimental studies with no clinical evidence of their involvement (Table 2).

**Figure 1:**
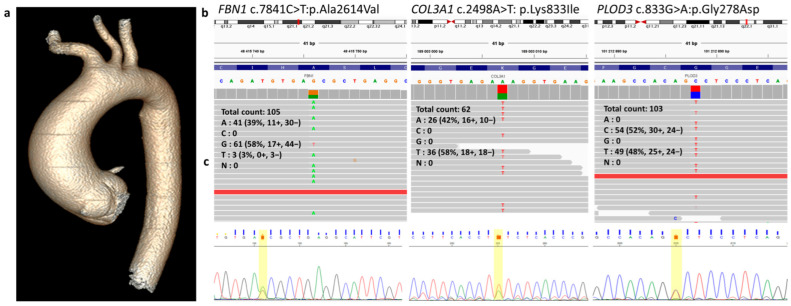
Identification of the c.7841C>T:p.Ala2614Val variant in FBN1, the c.2498A>T:p.Lys833Ile variant in COL3A1, and variant c.833G>A:p.Gly278Asp in PLOD3 in patient #54 with aneurysm of the ascending aorta. (**a**) MDCT of the thoracic aorta. (**b**) Molecular genetic testing of the patient by clinical exome sequencing. (**c**) Sanger sequencing validation of the variants. Genetic variants are highlighted in yellow.

Patient #54 (male, 48 years old, diameter of the mid-ascending aorta = 50 mm) was found to have three VUSs (in *FBN1*, *COL3A1*, and *PLOD3* genes; Table 2; Figure 1). Among the risk factors for TAAD and other cardiovascular diseases (CVDs), the patient received a diagnosis of arterial hypertension (Table 2). Additionally, he had a diagnosis of urolithiasis with microlith formation in the renal cavitary system of both kidneys.

*FBN1* (NC_000015.10) c.7841C>T (p.Ala2614Val) has never been previously identified in patients with TAAD. Potential pathogenicity or deleterious score of the variant was examined based on predictions of several in silico tools available at VarSome and CADD (PHRED; Table 2). Only the MutPred predictor available at VarSome assessed this variant as pathogenic, while nine other prediction scales assessed *FBN1* c.7841C>T as a VUS. Fifteen in silico tools available at VarSome evaluated *FBN1* c.7841C>T as benign. According to the CADD (PHRED) scale, this variant is pathogenic (23.6). Another variant—*FBN1* c.7841C>A p.(Ala2614Asp)—has been described previously at the same position of the gene (chr15:48415746) and was also classified as a VUS according to the VarSome pathogenicity scale (there is no information on allele frequency and disease association).

The second VUS, *COL3A1* (NC_000002.12) c.2498A>T (p.Lys833Ile), has not been described before, and its relation to this pathology is also unknown (Table 2). The VarSome pathogenicity prediction scale classifies this variant as pathogenic based on eight predictors (12: VUS, 3: benign). CADD (PHRED) also classified this variant as pathogenic (28.5). According to ClinVar, rs371344739 (*COL3A1* c.2498A>G, p.Lys833Arg, ID 199701) has previously been described at this position (chr2:189003007). The frequency of this VUS is described for populations in gnomAD (1.1 × 10^−4^).

The third VUS, *PLOD3* (NC_000007.14) c.833G>A (rs1041461490, p.Gly278Asp), is registered in gnomAD (6.8 × 10^−6^), RUSeq (5.9 × 10^−4^), and ClinVar (ID 1386463), but there is no information about its association with relevant pathologies (Table 2). CADD (PHRED) classifies this variant as pathogenic (25.6), although VarSome predictors classified this variant as pathogenic (11) or a VUS (10).

**Figure 2:**
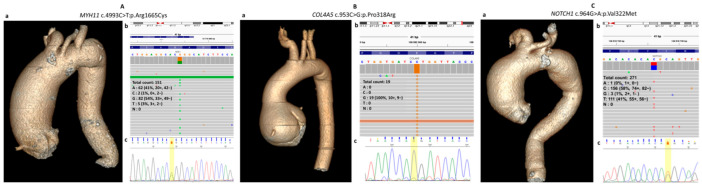
Identification of VUSs in a patient with aneurysm of the ascending aorta. (**A**) MDCT of the thoracic aorta in patient #43, results of molecular genetic testing of the patient by clinical exome sequencing, and Sanger sequencing validation of the c.4993C>T:p.Arg1665Cys variant in *MYH11*. (**B**) MDCT of the thoracic aorta in patient #13, results of molecular genetic testing of the patient by clinical exome sequencing, and Sanger sequencing validation of the c.953C>G:p.Pro318Arg variant in *COL4A5*. (**C**) MDCT of the thoracic aorta in patient #36, results of molecular genetic testing of the patient by clinical exome sequencing, and Sanger sequencing validation of the c.964G>A:p.Val322Met variant in *NOTCH1*. Genetic variants are highlighted in yellow.

Patient #43 (male, 72 years old, diameter of the mid-ascending aorta = 69 mm) was found to have a VUS: *MYH11* (NC_000016.10) c.4993C>T (rs768569707, p.Arg1665Cys) (Figure 2A). Along with nssTAA, this patient received a diagnosis of arterial hypertension, hyperlipidemia, atherosclerosis of coronary and carotid arteries, CAD, and chronic cerebral ischemia (Table 2). This patient had advanced coronary and aortic calcification (Agatston indices 423 and 937, respectively). Multicystic kidney, varicose veins, chronic gastritis, esophagitis, and osteochondrosis of the cervical spine were identified.

The frequency of the rs768569707 variant is 4.4 × 10^−5^ in gnomAD (Table 2). According to the VarSome pathogenicity prediction scale, this variant is pathogenic judging by 11 predictors (9: VUS and 1: benign); it is also categorized as pathogenic by the CADD (PHRED) scale (32.0). According to ClinVar, this variant (ID 405480) was previously reported in both patients with TAA and in children with left ventricular noncompaction, which is a hereditary type of cardiomyopathy [32].

Patient #13 (male, 58 years old, diameter of the mid-ascending aorta = 45 mm) has a VUS: *COL4A5* (NC_000023.11) c.953C>G (chrX:108582900, rs1449979085, p.Pro318Arg) (Figure 2B). This patient received a diagnosis of aortic root aneurysm, bicuspid aortic valve, arterial hypertension, hyperlipidemia, and obesity (Table 2). This patient also had stage 3b chronic kidney disease (glomerular filtration rate = 52 mL/min/1.73 m^2^).

The frequency of rs1449979085 variant is 8.3 × 10^−6^ among all populations in gnomAD (Table 2). According to VarSome, this variant is pathogenic based on three predictors (4: VUS, 16: benign); according to CADD (PHRED), this variant is closer to pathogenic (16.9). The VUS identified in patient #13 is described in ClinVar (ID 2485585), but information about its association with relevant pathologies, including TAA, is absent in the literature and publicly available databases.

VUS *NOTCH1* (NC_000009.12) c.964G>A (p.Val322Met) was identified in patient #36 (female, 67 years old, diameter of the mid-ascending aorta = 62 mm) (Figure 2C). She also received a diagnosis of dilation of the descending aorta, hypertension, paroxysmal tachycardia, CAD, carotid atherosclerosis, and chronic cerebral ischemia (Table 2). Low calcification of coronary arteries (Agatston index 49) and high calcification of the aorta (Agatston index 237) were detected.

Variant c.964G>A *NOTCH1* was detected with a frequency of 2.1 × 10^−6^ in gnomAD and is not registered in RUSeq population databases (Table 2). Among VarSome pathogenicity prediction scales, eight predictors classify this variant as pathogenic (10: VUS, 1: benign). Judging by CADD (PHRED), this variant is pathogenic (25.5). Previously, another VUS (c.964C>G, rs1317299348, p.Val322Leu) has been described in the NCBI database for these genome coordinates (chr9:136518726); however, there is no evidence that this variant is associated with a relevant disease. For *NOTCH1* c.964G>A, identified in the present study, there is no information about an association with any pathology either, including TAAD.

## 3. Discussion

In the current study, we identified variants of uncertain significance in Russian patients with nonsyndromic sporadic ascending aortic nondissecting aneurysm by massive parallel sequencing of genes associated with TAAD.

No pathogenic or likely pathogenic variants were found in the genes of thoracic aortic aneurysms. Nonetheless, we identified six VUSs. Of these, three VUSs [*FBN1* c.7841C>T (p.Ala2614Val), *COL3A1* c.2498A>T (p.Lys833Ile), and *MYH11* c.4993C>T (p.Arg1665Cys)] are in genes with a “definitive” effect of pathogenic variants on the pathology in question. Pathogenic variants in the *FBN1* gene are the main cause of Marfan syndrome (OMIM: 154700). Pathogenic variants in *COL3A1* are associated with vascular-type Ehlers–Danlos syndrome and with aneurysms of the aorta and other arteries (OMIM: 130050) [33]. Pathogenic variants in *MYH11* have been described in individuals having TAAD with patent ductus arteriosus (PDA) [34].

The remaining VUSs [*NOTCH1* c.964G>A (p.Val322Met), *COL4A5* c.953C>G (p.Pro318Arg), and *PLOD3* c.833G>A (p.Gly278Asp)] belong to genes with less evidence of the impact on TAAD. Nevertheless, pathogenic variants in the *COL4A5* gene can cause Alport syndrome (OMIM: 301050): a condition characterized by kidney disease, hearing loss, eye anomalies, and TAAD [35]. To date, data have been accumulating on the associations of *PLOD3* variants with connective tissue disease and its vascular complications as well as familial and sporadic vascular aneurysms in some populations [11,36,37]. It was demonstrated recently that *NOTCH1* mutations are associated with nssTAA in the absence of the bicuspid aortic valve [38].

The protein products of genes containing a VUS in patients with nssTAA without dissection are mainly structural components of the extracellular matrix (*FBN1*, *COL3A1*, and *COL4A5*). The other genes encode a major contractile protein of smooth muscle cells (*MYH11*), a transmembrane receptor of Notch signaling (*NOTCH1*), and an enzyme responsible for the stability of intermolecular collagen crosslinks (*PLOD3*). Of note, we did not find disease-causing variants in genes of the TGF-β signaling pathway, which plays a major role in TAAD pathogenesis [14]. This may in part be explained by our small sample size.

In our study, 9.8% of patients with nssTAA without dissection had ≥1 VUSs. Our results are in line with previous papers about mixed groups of TAAD patients (syndromic, familial, and sporadic cases) [3,21]. Nonetheless, the proportion of individuals with at least one observed VUS has been reported to be the highest (28%) in a subgroup of sporadic aortic dissection [9]. Moreover, in our work, all the identified VUSs were missense variants, consistent with a report on French patients with nonsyndromic thoracic aortic aneurysms and dissections, where the disease was associated with only missense variants in the *FBN1* gene [3].

Confirmation of a VUS’s relevance to sporadic ascending aortic aneurysm requires not only experimental studies but also clinical examination and genetic testing of first/second-degree relatives, especially for the TAAD cases with concomitant mutations in other genes. Further investigation is needed to address the family verification of the pathogenicity of these variants.

As new research results emerge confirming functional significance of genetic variants, pathogenicity criteria undergo changes, and variants may shift from one category to another [39]. On the other hand, some experimental research indicates that even VUSs that do not change their pathogenicity status with time can increase the risk of thoracic aortic aneurysm if combined with other genetic and nongenetic risk factors [40]. Therefore, we should keep patients with a VUS under observation including periodic imaging of the aorta starting from when the genetic variant was identified until the diagnosis is finally confirmed or refuted [19].

Genetic architecture of complex diseases (including nssTAA) is characterized by a combination of rare and common variants of genes. For some TAAD-causing genes, rare variants and common SNPs are associated not only with aortic aneurysm but also with other CVDs. For example, it has been shown that some variants of the *NOTCH1* gene are associated with aortic valve calcification [41]. A deletion affecting the *MYH11* gene has been identified in a family with pseudoxanthoma elasticum: a Mendelian disease featuring calcification of elastic fibers of the skin, arteries, and retina [42]. Common variants of the *MYH11* gene are associated with CAD and high blood pressure [43], and SNPs in the *FBN1* gene correlate with thoracic aortic aneurysm, coronary artery dissection, and blood pressure, according to the GWAS Catalog.

It is worth noting that in our study, patient #54 with three VUSs (in genes *FBN1*, *COL3A1*, and *PLOD3*) has only one CVD risk factor: arterial hypertension. VUSs in *MYH11* and *NOTCH1* were identified in two other patients here (#43 and #36), who have high calcification of coronary arteries and/or ascending aorta. Further investigation into both VUSs and analysis of common variants in the patients in combination with a deep clinical examination should help to identify relevant genotype–TAAD phenotype relations followed by the translation of these findings to clinical practice.

This study had several limitations. Firstly, the sample size was not large enough. Secondly, VUSs need further clinical family-based and functional validation. Thirdly, we analyzed only 53 genes associated with hereditary thoracic aortic aneurysm. However, there could be variants in other genes, that were not analyzed in this study. Exome or genome sequencing can help to overcome these shortcomings in a future study.

Despite the existing limitations of the study (small study group size), it may be reasonable to speculate that nssTAA without dissection shares the same molecular genetic mechanisms with syndromic and hereditary forms of TAAD. It has been shown that along with pathogenic and likely pathogenic variants, patients with sporadic aneurysm are characterized by accumulation of VUS, and GWAS studies have identified common variants in hereditary TAA and other genes [2,44].

Conclusion. The sporadic form is characterized by a unique genetic pattern, including different variants of a wide range of genes. Some of these variants may later be classified as pathogenic or benign, and some may be defined as a new class of rare “predisposing” low-penetrance variants, leading to the development of pathology in combination with other risk factors.

Future perspectives. To effectively reduce morbidity and mortality from thoracic aortic aneurysm/dissection in the future, it is necessary to identify this unique genetic pattern in patients with sporadic ascending aortic aneurysms. This task requires advanced genetic testing on an elaborated gene panel consisting of not only the 11 “causal” genes [2], but also genes that are assumed to be involved in hereditary and syndromic aneurysms. For some of these potential genes, their importance in the development of the pathology has not yet been confirmed by clinical studies. Confirmation of VUS significance in the development of sporadic ascending aortic aneurysm requires not only experimental studies, but also genetic testing of first/second-degree relatives. Moreover, we should provide clinical supervision for patients with potential causative variants including periodic imaging of the aorta starting from the moment when the genetic variant was identified until the diagnosis is finally confirmed or dismissed. Functional studies of VUSs, and population-level data with accurate phenotyping, will improve variant classification and reduce uncertainties for clinical implications of these variants.

## 4. Materials and Methods

### 4.1. Patient Characteristics

The study included patients with an aneurysm of the ascending aorta. All patients underwent open surgery at the Tomsk Cardiology Research Institute in the period from 2020 to 2023. All the candidates received a diagnosis of sporadic TAAD without any family history of aortic disease (their detailed characteristics are given in the Results Section 2). Patients were divided by sex (male/female) according to one assigned at birth.

Eligibility criteria were as follows: dilatation of the ascending aorta > 5.5 cm without aortic valve dysfunction or dilatation of the ascending aorta > 4.5 cm in the presence of severe aortic valve stenosis or insufficiency (grade 2+). Patients with aortic dissection/rupture, false aortic aneurysms, and patients requiring repeated interventions in the proximal thoracic aorta were excluded from the analysis.

Multidetector computed tomography (MDCT) was the basic method for diagnosis in patients with nssTAAD. The dimensions of the aorta were determined by MDCT perpendicular to the longitudinal axis, capturing the wall at several levels in accordance with national recommendations [45]. Image analysis and evaluation were performed independently by two experienced clinicians. MDCT of the aorta was performed on a 64-slice GE Discovery NM/CT 570C tomograph (GE Healthcare, Milwaukee, WI, USA) with the following parameters: 200–400 mA and 100–120 kW. Images were reconstructed with a slice thickness of 0.65 mm. A contrast agent was injected at a rate of 5 mL/s through the cubital vein by an automatic injector. The total volume of contrast was calculated on the basis of 1 mL per kilogram of body weight. To better visualize the aortic root and ascending part and to exclude artifacts, an ECG-synchronized analysis was performed from the bifurcation of the carotid arteries to the diaphragm. The resulting images were processed in Advantage Workstation 4.3 (GE Healthcare, USA).

Comorbidities were confirmed based on medical history and instrumental examination, including duplex ultrasonography of carotid, subclavian, vertebral, and femoral arteries; echocardiography; and coronary angiography. All patients underwent primary medical and genetic counseling, which included family history taking with pedigree construction, physical examination, and tests for congenital connective tissue dysplasia (assessed using Marfan and Beighton scores) [46,47].

### 4.2. Virtual Gene Panel Analysis

We searched for rare genetic variants (minor allele frequency < 1%) located in the exons of 53 genes associated with hereditary thoracic aortic aneurysm. The selection of genes was based on the data present in the ClinGen database (Appendix A) and in the literature (Supplementary Table S2) [10,13,17,29,48,49].

### 4.3. Clinical Exome Sequencing

DNA was isolated from peripheral blood leukocytes by phenol–chloroform extraction [50]. DNA was dissolved in TE Buffer, Tris-EDTA, 1 x Solution, pH 8.0. The quality and quantity of the DNA were checked by means of a Nanodrop 8000 spectrophotometer (Thermo, Waltham, MA, USA), a Qubit 3.0 fluorimeter (Thermo, USA), and a 1% LE agarose gel with 1× Tris-Acetate-EDTA (TAE) Buffer, pH 8.0.

DNA libraries were prepared using the Clinical Exome Solution Kit (Sophia Genetics, Lausanne, Switzerland), KAPA Library Amplification Kit KK2620 (Roche, Munich, Germany), and Agencourt AMPure XP magnetic beads (Beckman Coulter, Brea, CA, USA) according to the Clinical Exome Solution protocol (Sophia Genetics, Switzerland). The libraries were then quantified on a Qubit 3.0 fluorimeter (Thermo, Waltham, MA, USA) and a Bioanalyzer 2100 capillary electrophoresis system (Agilent, Santa Clara, CA, USA).

Sequencing was performed on the NextSeq 500 Illumina platform (Illumina, San Diego, CA, USA) with NextSeq 500/550 Mid Output Kit v.2 (300 cycles in 150 bp paired-end mode). Between 97% and 98.1% of the sequence reads were then successfully aligned to the reference genome (GRCh38) using Illumina DRAGEN Bio-IT. Exons of 53 target genes, representing 55% of the total exon length, had a minimum coverage of 30× in 79.3% (ranging between 77.87% and 80.4%) of cases, with an average coverage of 66.5×.

Variant annotation was performed in ANNOVAR. Classification of identified variants by degree of pathogenicity was carried out according to the American College of Medical Genetics (ACMG) standards and guidelines for the interpretation of sequence variants. For in silico pathogenicity prediction, we used VarSome, The Human Genomics Community tool (Appendix A). This is an automated classifier of genetic variants by degree of pathogenicity based on several in silico tools, including CADD, Polyphen2 HDIV, Polyphen2 HVAR, DEOGEN2, and PROVEAN. The frequency of VUS was determined using gnomAD (v4.1.0) and RUSeq Browser (Appendix A).

### 4.4. Sanger Sequencing

Validation of clinically relevant variants identified in genes *FBN1*, *MYH11*, *COL3A1*, *COL4A5*, *NOTCH1*, and *PLOD3* was performed by bidirectional Sanger Sequence analysis on a 3730xl DNA Analyzer (Applied Biosystems, Norwalk, CT, USA). PCR amplification was performed using ready-to-use reaction mix HS-Taq PCR-Color (2×) (Biolabmix, Novosibirsk, Russia) with the primers specified in Table 3.

The specific primers were designed according to the hg38 genome assembly by means of the Primer Quest Tool and Primer Design and Search Tool (Appendix A).

The following reagents were used for purification of PCR products: thermolabile exonuclease Exo I (Thermo Scientific, Waltham, MA, USA), shrimp alkaline phosphatase (Thermo Scientific, USA), and deionized water.

The subsequent sequencing reaction was carried out using the BigDye Sequencing Kit v3.1 (Thermo Scientific, USA), 5× sequencing buffer, a forward or reverse primer, PCR product, and deionized water. Sequencing reaction products were purified with the D-Pure Reagent Kit (Nijmegen, The Netherlands). Sanger DNA sequencing result visualization and analysis were performed in the UGENE software (v42.0, Unipro, Moscow, Russia) [51].

### 4.5. Statistics

The normality of distribution was checked using the Shapiro–Wilk test. Group comparison was performed using non-parametric tests (Mann–Whitney). Results are presented as median, 1st and 3rd quartiles (Me (Q1; Q3)). For the analysis of qualitative parameters, Fisher’s exact test was applied. Using the PVR package in R, we also calculated the minimum effect size that can be detected at a power of 0.8. Differences were considered significant at *p* < 0.05.

## Figures and Tables

**Table 1:** Description of patients included in the study.

Parameters	All Patients (n = 41)	Males (n = 27)	Females (n = 14)	*p*-Value
Age, years (Median (Q1; Q3))	54(48; 64)	58.0 (48.0; 62.5)	53.0 (48.8; 68.5)	0.563
Mid-ascending aorta diameter, mm (Median (Q1; Q3))	52 (49; 54)	51.9 (46.5; 54.0)	52.0 (50.3; 52.9)	0.535
Sinotubular junction diameter, mm (Median (Q1; Q3))	42 (38.2; 46)	44.0 (39.9; 52.3)	39.0 (37.0; 40.9)	**0.012**
Proximal aortic arch diameter, mm (Median (Q1; Q3))	39.5 (36; 42.5)	39.5 (36.0; 41.8)	39.5 (36.0; 43.0)	0.976
Bicuspid aortic valve, n (%)	25 (61.0)	14 (51.8)	11 (78.6)	0.105
BMI, kg/m^2^ (Median (Q1; Q3))	29 (26.4; 31.6)	29 (27.3; 31.4)	29.1 (23.6; 32.3)	0.530
Arterial hypertension, n (%)	27 (64.3)	18 (64.3)	9 (64.3)	0.879
Hyperlipidemia, n (%)	10 (23.8)	7 (25.0)	3 (21.4)	0.751
Obesity (BMI (kg/m^2^) > 30), n (%)	18 (42.9)	12 (42.9)	6 (42.9)	1
T2DM or glucose intolerance, n (%)	3 (7.1)	2 (7.1)	1 (7.1)	1
Angina pectoris, CAD or MI, n (%)	13 (30.9)	12 (42.8)	1 (7.1)	**0.039**
Atherosclerosis, coronary, carotid, renal or femoral, n (%)	21 (50)	16 (59.2)	4 (28.6)	0.055
Calcification measurements (number of patients)	26	18	8	-
Ca-score in coronary arteries (Agatston index)	71.2	94.9	17.9	0.414
Ca-score in aorta (Agatston index)	107	121	75.5	0.404

BMI: body–mass index, Ca-score: calcium score (Agatston index), MI: myocardial infarction, Q1: lower quartile, Q3: upper quartile, and *p*-value indicates the level of significance obtained from comparisons between men and women. The criterion for hyperlipidemia was the excess of optimal lipid values (LHD, LDL, triglycerides) depending on the risk profile of the patient. The diagnosis of arterial hypertension was based on stably elevated blood pressure at repeated (multiple) measurements on different days and according to the history of hypertension. Values *p* < 0.05 are highlighted in bold.

**Table 2:** Characteristics of patients with a VUS(s).

Characteristics ID		Patient 13	Patient 36	Patient 43	Patient 54		
Age (years)		58	67	72	48		
Sex		m	f	m	m		
Gene (category, (13))		*COL4A5* (C)	*NOTCH1* (B)	*MYH11* (A)	*FBN1* (A)	*COL3A1* (A)	*PLOD3* (D)
Genome coordinates (hg38/exon)		chrX: 108582900/ exon 17	chr9: 136518726/ exon 6	chr16: 15719674/ exon 35	chr15: 48415746/ exon 64	chr2: 189003007/ exon 36	chr7: 101212888/ exon 8
Nucleotide:aminoacid change		c.953C>G: p.Pro318Arg	c.964G>A: p.Val322Met	c.4993C>T: p.Arg1665Cys	7841C>T: p.Ala2614Val	c.2498A>T: p.Lys833Ile	c.833G>A: p.Gly278Asp
SNP ID MAF *		rs1449979085 8.3 × 10^−6^/-	- 2.1 × 10^−6^/-	rs768569707 4.4 × 10^−5^/-	- 6.8 × 10^−7^/-	- -	rs1041461490 6.8 × 10^−6^/5.9 × 10^−4^
In silico predictors **		3/4/16 16.9	8/10/1 25.0	11/9/1 32.0	1/7/15 23.6	8/12/3 28.5	0/1/0 25.6
Aortic dimensions ***		70/45/45/34	36/62/45/35	41/69/48/36	45/50/35/30		
Ca-score (Agatston index)	Coronary arteries	no data	49	423	0		
	Aorta	no data	237	937	0		
Aortic valve		BAV	TAV	TAV	TAV		
CAD		no	yes	yes	no		
CVD risk factors (HTN/T2DM/HLD/O)		yes/no/yes/yes	yes/no/-/no	yes/no/yes/no	yes/no/no/no		
Atherosclerosis		no	CarA:up to 20%	CorA: OMA 60%; LAD 40%; DA 40%; CarA: up to 20%	no		

BAV: bicuspid aortic valve, DA: diagonal branches, f: female, HLD: hyperlipidemia, HTN: hypertension, LAD: left anterior descending artery, m: male, MAF: minor allele frequency, O: obesity, OMA: obtuse marginal artery, SNP: single-nucleotide polymorphism, TAV: tricuspid aortic valve, CorA: coronary arteries, CarA: carotid arteries. On the basis of the strength of association with HTAAD, genes were classified into categories: A, definitive; B, strong; C, moderate; or D, limited [13]. MAF *—gnomAD total v. 4.1.0/RUSeq. In silico Predictors **—VarSome: Pathogenic/Uncertain/Benign; CADD (PHRED). Aortic dimensions ***—sinotubular junction/mid-ascending aorta/proximal aortic arch/arch (mm, according to MDCT).

**Table 3:** Details of the genes and primers used for Sanger sequencing.

Gene	Exon	Sequence
*COL3A1* NC_000002.12	36	F: 5′-GCTGAGAGATTGCTGTTG-3′ R: 5′-GGTGCTGAGATTCATACTTG-3′
*FBN1* NC_000015.10	64	F: 5′-GACAGCCACACAGGTAA-3′ R: 5′-CATAGCAAGAAGCCACATC-3′
*MYH11* NC_000016.10	35	F: 5′-CAGAGGAGGACGAAATGA-3′ R: 5′-TGTGCAAAGCTGAACTG-3′
*COL4A5* NC_000023.11	17	F: 5′-CCAGTATTCTCATTGCTTCTAT-3′ R: 5′-TATTTCTGCAACATGGACTG-3′
*NOTCH1* NC_000009.12	6	F: 5′-GGACACTCGCAGTAGAA-3′ R: 5′-TCCACAGAGCACAAAGA-3′
*PLOD3* NC_000007.14	8	F: 5′-GCTGGAAGATGCAACAC-3′ R: 5′-GGAAACGGTCCCACTAA-3′

## Data Availability

Dataset available upon request from the authors.

## Supplementary Materials

The following supporting information can be downloaded at: https://www.mdpi.com/article/10.3390/ijms25158315/s1.

## Appendix A

GWAS catalog:
https://www.ebi.ac.uk/gwas/search?query=thoracic%20aortic%20aneurysm, accessed on 15 January 2023
https://www.ebi.ac.uk/gwas/genes/MYH11, accessed on 20 June 2024
https://www.ebi.ac.uk/gwas/genes/FBN1, accessed on 20 June 2024
ClinGen: https://clinicalgenome.org/, accessed on 15 January 2023
VarSome The Human Genomics Community: https://varsome.com/, accessed on 20 June 2024
PrimerQuest Tool: https://eu.idtdna.com/pages, accessed on 8 April 2023
Primer Design and Search Tool: http://bisearch.enzim.hu/, accessed on 8 April 2023
NCBI:
https://www.ncbi.nlm.nih.gov/snp/rs1317299348, accessed on 20 June 2024
https://www.ncbi.nlm.nih.gov/snp/rs371344739, accessed on 20 June 2024
CADD: Combined Annotation Dependent Depletion: https://cadd.gs.washington.edu/snv, accessed on 20 June 2024
gnomAD browser, v. 4.1.0: https://gnomad.broadinstitute.org, accessed on 20 June 2024
RUSeq Browser: http://ruseq.ru/, accessed on 20 June 2024
PVR package in R: https://cran.r-project.org/web/packages/pwr/, accessed on 1 February 2023
