# Supplementary material for: Identification of Variants of Uncertain Significance in the Genes Associated with Thoracic Aortic Disease in Russian Patients with Nonsyndromic Sporadic Subtypes of the Disorder
Source: Int J Mol Sci. 2024 Jul 30;25(15):8315. doi: 10.3390/ijms25158315 (PMC11312146; doi:10.3390/ijms25158315)
Supplement: Supplementary file 1 [file ijms-25-08315-s001.zip › ijms-3091155-SI.pdf]

Supplementary Information

Identification of variants of uncertain significance in the genes associated with thoracic aortic disease in Russian patients with nonsyndromic sporadic subtypes of the disorder

Irina A. Goncharova, Sofia A. Shipulina, Aleksei A. Sleptcov, Aleksei A. Zarubin, Nail R. Valiakhmetov, Dmitry S. Panfilov, Evgeniya V. Lelik, Viktor V. Saushkin,

Boris N. Kozlov, Ludmila P. Nazarenko, Maria S. Nazarenko

Table S1: Genetic variants identified in the genes of hereditary aneurysms in Russian patients with sporadic aneurysms of the ascending aorta.

Chr	Ref	Alt	Gene Symbol	Interpretion	rsID	MAF (GnomAD)	Sample	Sex	Age	Aortic dimensions (mm, according to MDCT		
										sinotubular junction	mid-ascending aorta	proximal aortic arch
chr2:189003007	A	T	*COL3A1*	Uncertain Significance	.							
chr15:48415746	G	A	*FBN1*	Uncertain Significance	.							
chr7:101212888	C	T	*PLOD3*	Uncertain Significance	rs1041461490		54	Male	48	45	50	35
chr5:128527886	G	A	*FBN2*	Benign	rs147157552	0,0012						
chr9:136523963	C	T	*NOTCH1*	Benign	rs757497167	3,97E-05						
chr9:134700009	G	T	*COL5A1*	Benign	rs145178917	0,0037	27	Male	74	44	52	44
chr16:15717308	G	A	*MYH11*	Benign	rs201960644	0,0006						
chr7:74063312	G	A	*ELN*	Benign	rs150404125	0,0011	55	Female	74	37	52	35
chr16:15719674	G	A	*MYH11*	Uncertain Significance	rs768569707	5,17E-05						
chr14:75971659	A	C	*TGFB3*	Benign	rs201453600	0,0001	43	Male	72	41	69	48
chr3:123700244	A	G	*MYLK*	Benign	.	.						
chr3:123700257	C	T	*MYLK*	Benign	rs752986671	1,99E-05	23	Male	50	46	50	36
chr3:123739976	C	A	*MYLK*	Benign	rs140148380	0,0018						
chr5:83539770	T	C	*VCAN*	Benign	rs146630369	0,0007	40	Male	59	40	49	41
chr21:45476433	C	T	*COL18A1*	Likely Benign	.	.						
chr3:30623222	G	A	*TGFBR2*	Benign	rs61732532	0,0014	7	Female	77	40,4	52,6	45
chr5:122077510	G	T	*LOX*	Benign	rs41407546	0,0057						
chr21:45456740	G	A	*COL18A1*	Benign	rs141363853	0,0036	48	Male	52	35	47	36
chr11:65871991	G	A	*EFEMP2*	Benign	rs144320036	0,001	14	Female	51	44,6	52,7	36

chr15:73322516	C	G	*HCN4*	Benign	rs200507617	0,0007						
chrX:108582900	C	G	*COL4A5*	Uncertain Significance	.	5,46E-06						
chr1:11944569	C	T	*PLOD1*	Benign	rs75220940	0,0047	13	Male	58	70	45	45
chr9:136518726	C	T	*NOTCH1*	Uncertain Significance	.	.						
chr19:8601022	C	T	*ADAMTS10*	Benign	rs144166844	0,0006	36	Female	67	36	62	45
chr1:102962756	G	T	*COL11A1*	Likely Benign	rs78046647	0,0017						
chr20:62475514	T	C	*GATA5*	Benign	rs113068438	0,0058	37	Male	29	44,9	53	34,3
chr21:45456333	T	C	*COL18A1*	Likely Benign	.	.						
chr20:10641566	C	T	*JAG1*	Benign	rs145895196	0,0019	53	Female	38	37	49	43
chr4:88067937	A	C	*PKD2*	Benign	rs2234917	0,0049	49	Male	61	42	55	35
chr4:88008136	G	A	*PKD2*	Benign	rs886059697	0,0001	42	Male	51	59,3	44,8	36,6
chr1:218346973	G	A	*TGFB2*	Benign	rs10482721	0,004	29	Male	67	43	51	36
chr11:65871247	C	T	*EFEMP2*	Benign	rs2234462	0,0032	32	Female	54	51	61	38
chr1:102888579	G	C	*COL11A1*	Benign	rs139064549	0,0086	39	Female	48	40	53	39,5
chrX:154359007	T	C	*FLNA*	Benign	rs200130356	0,001	30	Male	49	54,5	51,9	38,1
chr21:45456848	G	A	*COL18A1*	Benign	rs545040488	0,0026	26	Male	37	50	54,8	40
chr21:45456302	C	T	*COL18A1*	Likely Benign	rs561076797	3,24E-05	44	Male	60	38,2	53	41
chr9:134817038	C	T	*COL5A1*	Benign	rs61739195	0,0075	38	Female	44	39	52	41
chr9:134809214	G	A	*COL5A1*	Likely Benign	rs759580799	5,94E-05	9	Male	48	58,5	79	34,4
chr9:134750815	C	T	*COL5A1*	Benign	rs369000939	3,6E-05	50	Female	74	38	51	39
chr1:40315652	C	T	*COL9A2*	Benign	rs193238892	0,0001	22	Male	48	39,8	51,2	26,2
chr20:10640917	C	T	*JAG1*	Benign	rs376089631	2,39E-05	20	Male	62	35	47	36
chr15:66703431	C	G	*SMAD6*	Benign	rs756752655	0,0004	23, 30	See above	See above	See above	See above	See above
chr17:50188134	C	T	*COL1A1*	Benign	rs1800215	.	37,39	See above	See above	See above	See above	See above
chr21:45509458	G	A	*COL18A1*	Benign	rs113268527	0,0066	9,32,44	See above	See above	See above	See above	See above
							4	Male	46	39,6	52	40,4
							8	Male	32	31	45	46
							17	Male	68	40	42	44
							21	Male	64	65,4	43,4	41,1
							28	Male	58	47,8	70,7	41,1
chr19:8605046	C	G	*ADAMTS10*	Benign	rs7255721	-	41	Female	52	41	45	34

							14, 20, 23,26, 29,32,35, 37,42, 48, 49, 53	See above	See above	See above	See above	See above

Table S2: Genes for hereditary and syndromic forms of thoracic aortic aneurysm.

Gene Symbol	Loci	The strength of the association of genes with HTAA (PMID:30071989/ClinGene 11.01.2023)	Gene product	OMIM id	Sourse
ACTA2	10q23.31	Definitive/Definitive	Actin alpha 2	Aortic aneurysm familial thoracic, 6 (#611788)	PMID:30071989/ClinGene 11.01.2023
FBN1	15q21.1	Definitive/Definitive	Fibrillin-1	Marfan syndrome (#154700)	PMID:30071989/ClinGene 11.01.2024
MYH11	16p13.11	Definitive/Definitive	Myosin Heavy Chain 11	Aortic Aneurysm familial thoracic, 4 (#132900)	PMID:30071989/ClinGene 11.01.2025
SMAD3	15q22.33	Definitive/Definitive	SMAD Family Member 3	Loeys-Dietz syndrome 3 (#613795)	PMID:30071989/ClinGene 11.01.2026
TGFB2	1q41	Definitive/Definitive	Transforming Growth Factor Beta 2	Loeys-Dietz syndrome 4 (#614816)	PMID:30071989/ClinGene 11.01.2027
TGFBR1	9q22.33	Definitive/Definitive	Transforming Growth Factor Beta Receptor 1	Loeys-Dietz syndrome 1 (#609192)	PMID:30071989/ClinGene 11.01.2028
TGFBR2	3p24.1	Definitive/Definitive	Transforming Growth Factor Beta Receptor 2	Loeys-Dietz syndrome 2 (#610168)	PMID:30071989/ClinGene 11.01.2029
MYLK	3q21.1	Definitive/Strong	Myosin Light Chain Kinase	Aortic aneurysm familial thoracic, 7 (#613780)	PMID:30071989/ClinGene 11.01.2030
LOX	5q23.1	Strong/Strong	Lysyl Oxidase	Aortic aneurysm familial thoracic, 10 (#617168)	PMID:30071989/ClinGene 11.01.2031
PRKG1	10q11.23-q21.1	Strong/Strong	Protein Kinase CGMP-Dependent 1	Aortic aneurysm familial thoracic, 8 (#615436)	PMID:30071989/ClinGene 11.01.2032
COL3A1	2q32.2	Definitive/ no	Collagen Type III Alpha 1 Chain	Ehlers-Danlos syndrome type IV (#130050)	PMID:30071989/ClinGene 11.01.2033
EFEMP2	11q13.1	Moderate/Moderate	EGF Containing Fibulin Extracellular Matrix Protein 2	Cutis Laxa, Autosomal Recessive, Type 1A&B (#614437)	PMID:30071989/ClinGene 11.01.2034
FOXE3	1p33	Uncertain/Moderate	Forkhead Box E3	Aortic Aneurysm Familial Thoracic, 11 (#617349)	PMID:30071989/ClinGene 11.01.2035
SMAD2	18q21.1	Uncertain/Moderate	SMAD Family Member 2	no	PMID:30071989/ClinGene 11.01.2037

HCN4	15q24.1	Uncertain/ Limited	Hyperpolarization Activated Cyclic Nucleotide Gated Potassium Channel 4	no	PMID:30071989/ClinGene 11.01.2038
TGFB3	14q24.3	Uncertain/ Limited	Transforming Growth Factor Beta 3	Loeys-Dietz syndrome 5(#615582)	PMID:30071989/ClinGene 11.01.2040
BGN	Xq28	Uncertain/Limited	Biglycan	Meester-Loeys syndrome (#300989)	PMID:30071989/ClinGene 11.01.2041
FBN2	5q23.3	Limited/ Limited	Fibrillin 2	Contractural Arachnodactyly, Congenital (#121050)	PMID:30071989/ClinGene 11.01.2042
FLNA	Xq28	Limited/ Limited	Filamin A	Periventricular Nodular Heterotopia (#300049)	PMID:30071989/ClinGene 11.01.2043
NOTCH1	9q34.3	Limited/ Limited	Notch homolog 1, translocation-associated	Aortic Valve Disease 1(#109730)	PMID:30071989/ClinGene 11.01.2044
SLC2A10	20q13.12	Limited/ Limited	Solute carrier family 2, facilitated glucose transporter member 10	Arterial Tortuosity Syndrome (#208050)	PMID:30071989/ClinGene 11.01.2045
CBS	21q22.3	Limited/ no	Cystathionine Beta-Synthase	Homocystinuria (#236200)	PMID:30071989/ClinGene 11.01.2046
COL4A5	Xq22.3	Limited/ no	Collagen Type IV Alpha 5 Chain	Alport Syndrome, X-linked (#301050)	PMID:30071989/ClinGene 11.01.2047
ELN	7q11.23	Limited/ no	Elastin	Cutis Laxa, Autosomal Dominant 1 (#123700)	PMID:30071989/ClinGene 11.01.2048
PKD1	16p13.3	Limited/ no	Polycystin 1, Transient Receptor Potential Channel Interacting	Polycystic Kidney Disease 1 (#173900)	PMID:30071989/ClinGene 11.01.2049
PKD2	4q22.1	Limited/ no	Polycystin 2, Transient Receptor Potential Cation Channel	Polycystic Kidney Disease 2 (#613095)	PMID:30071989/ClinGene 11.01.2050
SKI	1p36.33-p36.32	Limited/ no	SKI Proto-Oncogene	Shprintzen-Goldberg Craniosynostosis syndrome (#182212)	PMID:30071989/ClinGene 11.01.2051
SMAD4	18q21.2	Limited/ no	SMAD Family Member 4	Juvenile polyposis syndrome (#174900); Hereditary hemorrhagic telangiectasia(#17505)	PMID:30071989/ClinGene 11.01.2052
COL9A1	6q13	No evidence/ Limited	Collagen Type IX Alpha 1 Chain	Stickler syndrome, type IV (#614134)	PMID:30071989/ClinGene 11.01.2053
ADAMTS10	19p13.2	No evidence/ no	ADAM Metallopeptidase	Weill-Marchesani syndrome	PMID:30071989/ClinGene

			With Thrombospondin Type 1 Motif 10	(#277600)	11.01.2055
B3GAT3	11q12.3	No evidence/ no	Beta-1,3-Glucuronyltransferase 3	Multiple Joint Dislocation, Short Stature, and Craniofacial Dysmorphism With Or Without Congenital Heart Defects (#245600)	PMID:30071989/ClinGene 11.01.2056
COL11A1	1p21.1	No evidence/ no	Collagen Type XI Alpha 1 Chain	Stickler syndrome, type II (#604841)	PMID:30071989/ClinGene 11.01.2057
COL18A1	21q22.3	No evidence/ no	Collagen Type XVIII Alpha 1 Chain	Knobloch syndrome, type 1 (#267750)	PMID:30071989/ClinGene 11.01.2058
COL1A1	17q21.33	No evidence/ no	Collagen Type I Alpha 1 Chain	Osteogenesis Imperfecta Type 1 (#166200)	PMID:30071989/ClinGene 11.01.2059
COL1A2	7q21.3	No evidence/ no	Collagen Type I Alpha 2 Chain	Ehlers-Danlos syndrome, cardiac valvular form (#225320)	PMID:30071989/ClinGene 11.01.2060
COL4A1	13q34	No evidence/ no	Collagen Type IV Alpha 1 Chain	Angiopathy, hereditary, with nephropathy, aneurysms, and muscle cramps (#611773)	PMID:30071989/ClinGene 11.01.2061
COL5A1	9q34.3	No evidence/ no	Collagen Type V Alpha 1 Chain	Ehlers-Danlos syndrome, classic type (#130000)	PMID:30071989/ClinGene 11.01.2062
COL5A2	2q32.2	No evidence/ no	Collagen Type V Alpha 2 Chain	Ehlers-Danlos syndrome, classic type (#130000)	PMID:30071989/ClinGene 11.01.2063
COL9A2	1p34.2	No evidence/ no	Collagen Type IX Alpha 2 Chain	Stickler syndrome, type V (#614284)	PMID:30071989/ClinGene 11.01.2064
ENG	9q34.11	No evidence/ no	Endoglin	Familial cerebral saccular aneurysm (231160 - Orphanet id)	PMID:30071989/ClinGene 11.01.2066
GATA5	20q13.33	No evidence/ no	GATA Binding Protein 5	Rare disease with thoracic aortic aneurysm and aortic dissection (285014 - Orphanet id)	PMID:30071989/ClinGene 11.01.2067
JAG1	20p12.2	No evidence/ no	Jagged Canonical Notch Ligand 1	Alagille syndrome (#118450)	PMID:30071989/ClinGene 11.01.2069
MED12	Xq13.1	No evidence/ no	Mediator Complex Subunit 12	Lujan-Fryns syndrome (#309520)	PMID:30071989/ClinGene 11.01.2070
PLOD1	1p36.22	No evidence/ no	Procollagen-Lysine,2-Oxoglutarate 5-Dioxygenase 1	Ehlers-Danlos syndrome, type VI (#225400)	PMID:30071989/ClinGene 11.01.2071
PLOD3	7q22.1	No evidence/ no	Procollagen-Lysine,2-	Lysyl hydroxylase 3 deficiency	PMID:30071989/ClinGene

			Oxoglutarate 5-Dioxygenase 3	(#612394)	11.01.2072
SMAD6	15q22.31	No evidence/ no	SMAD Family Member 6	Aortic valve disease 2 (#614823)	PMID:30071989/ClinGene 11.01.2073
UPF3B	Xq24	No evidence/ no	UPF3B Regulator Of Nonsense Mediated MRNA Decay	X-linked intellectual disability with marfanoid habitus (776 - Orphanet id)	PMID:30071989/ClinGene 11.01.2074
VCAN	5q14.2-q14.3	No evidence/ no	Versican	no	PMID:30071989/ClinGene 11.01.2075
LTBP3	11q13.1	no/no	Latent transforming growth factor-beta-binding protein 3	DASS dental anomalies and short stature (# 601216)	PMID: 36044906; PMID: 29625025
FKBP14	7p14.3	no/no	FK506-binding protein 14	Ehlers-Danlos syndrome, kyphoscoliotic type, 2 (#614557)	PMID: 36044906
ABL1	9q34.12	no/no	ABL protooncogene 1	Congenital heart defects and skeletal malformations syndrome (#617602)	PMID: 36044906
EP300	22q13.2	no/no	E1A-binding protein	Colorectal cancer, somatic (#114500); Menke-Hennekam syndrome 2 (#618333); Rubinstein-Taybi syndrome 2 (#613684)	PMID: 36044906; PMID: 33083483
KCNMA1	10q22.3	no/no	Potassium channel, calcium-activated, large conductance, subfamily m, alpha member 1	Cerebellar atrophy, developmental delay, and seizures (#617643); Liang-Wang syndrome (#618729); Paroxysmal nonkinesigenic dyskinesia, 3, with or without generalized epilepsy (609446); {Epilepsy, idiopathic generalized, susceptibility to, 16 (618596)	PMID: 36044906

Table S3: Characteristics of patients without VUSs.

Sample	Age	Sex	Aortic dimensions (mm, according to MDCT)			Ca-score (Agatston index)		Aortic valve	CAD	Hypertension	T2DM	Hyperlipidemia	Obesity	Atherosclerosis
			Sinotubular junction	Mid-ascending aorta	Proximal aortic arch	Coronary arteries	Aorta							
2	61	f	39	52		no data	no data	BAV	no	yes	no	yes	yes	no
3	51	f	43	50	32	no data	no data	BAV	no	yes	no	no	no	no
4	46	m	39,6	52	40,4	28	0	BAV	yes	no	no	no	yes	no
7	77	f	40,4	52,6	45	no data	no data	TAV	no	yes	no	no data	yes	CarA 20%
8	32	m	31	45	46	no data	no data	BAV	no	no	no	no data	yes	no
9	48	m	58,5	79	34,4	no data	no data	TAV	no	yes	no	no data	yes	no
12	65	m	46	54	42	no data	no data	BAV	no	no	no	no data	yes	no
14	51	f	44,6	52,7	36	no data	no data	BAV	no	no	no	no data	no	no
15	53	m	55	52	41	no data	no data	TAV	yes	yes	no	yes	yes	CorA
16	63	m	37	41	no data	no data	no data	TAV	no	yes	no	no data	no	renal
17	68	m	40	42	44	no data	no data	TAV	yes	yes	no	no data	no	CorA
20	62	m	35	47	36	8	301	BAV	yes	no	no	yes	no	CorA
21	64	m	65,4	43,4	41,1	311	85	TAV	yes	yes	yes	no data	yes	CarA 20%
22	48	m	39,8	51,2	26,2	80	0	TAV	yes	yes	no	no data	yes	CorA
23	50	m	46	50	36	0	5	BAV	yes	yes	no	no data	no	CorA, CarA
26	37	m	50	54,8	40	0	0	BAV	no	no	no	no	no	no
27	74	m	44	52	44	581	269	TAV	no	no	no	no data	no	CorA, CarA
28	58	m	47,8	70,7	41,1	102	41	TAV	yes	yes	no	no data	no	CorA: RCA 65%; OMA 60%; LAD 30%; CarA 20%; femoral arteries 5%

29	67	m	43	51	36	159	0	BAV	yes	yes	no	yes	no	CorA: LAD 75%; RCA 25%;CarA 40%
30	49	m	54,5	51,9	38,1	16	202	BAV	no	no	no	yes	no	CorA: LAD 50%; femoral arteries 15%
31	69	f	32	54	43	28	0	TAV	no	yes	no	no data	yes	no
32	54	f	51	61	38	0	0	BAV	no	yes	no	yes	yes	no
33	62	m	42	46	39	no data	no data	TAV	yes	yes	no	yes	yes	CorA: DA 75%; LAD 30%; OMA 30%; CarA 30%; femoral arteries 10%
35	47	m	64	54	37,5	0	0	BAV	no	yes	no	no data	yes	no
37	29	m	44,9	53	34,3	0	0	BAV	no	no	no	no	no	no
38	44	f	39	52	41	0	0	BAV	no	no	no	no data	no	no
39	48	f	40	53	39,5	0	0	BAV	no	no	no	no data	no	no
41	52	f	41	45	34	no data	no data	BAV	no	no	no	no	no	no
42	51	m	59,3	44,8	36,6	no data	no data	TAV	no	yes	no	no	yes	CorA: ПКА 30%
44	60	m	38,2	53	41	0	0	BAV	no	yes	no	no	no	no
47	60	m	44	55	43	0	331	mechanical prosthesis	no	yes	glucose intolerance	no	yes	CarA 20%
48	52	m	35	47	36	0	7	BAV	no	no	no	no	no	no
49	61	m	42	55	35	0	0	BAV	no	yes	no	no	no	CorA: LDA 30%
50	74	f	38	51	39	66	367	BAV	no	yes	no	yes	no	CorA: DA 40%; LDA 30%; CarA

														25%; femoral arteries 20%
52	48	f	35,8	50	40,2	0	0	BAV	no	no	no	no	yes	no
53	38	f	37	49	43	0	0	BAV	no	yes	no	no	yes	no
55	74	f	37	52	35	no data	no data	BAV	no	yes	glucose intoleranc e	no data	no	CorA: LDA 30%;CarA - 25%; femoral arteries 25%
